# Systemic Sporotrichosis With Testicular Involvement: Literature Review and Case Report

**DOI:** 10.1155/criu/4235866

**Published:** 2025-10-03

**Authors:** Juan Eduardo Rios Rodriguez, Andreia Naiuf Lima Tuma, Aiko Iwamoto, Déborah Cristina Andrade Neves, Alexandre Kapteinat Lima, Felipe Nogueira Clementoni, Paula Heroso Moreira, Juan Felipe Martins Filgueiras, Luiz Sergio Santos, Osny de Barros Junior

**Affiliations:** ^1^Department of Urology, Clinical Hospital Complex of the Federal University of Paraná (CHC-UFPR), Curitiba, Paraná, Brazil; ^2^Medical School, Federal University of Paraná (UFPR), Curitiba, Paraná, Brazil

**Keywords:** alcoholism, case report, HIV, immunocompromised host, *Sporothrix* sp., testicle

## Abstract

**Introduction:**

Sporotrichosis is an endemic fungal infection in Brazil, caused by a dimorphic fungus of the genus *Sporothrix*. Transmission occurs through traumatic inoculation from soil, contaminated plants, and zoonotic sources, mainly from cats, as well as through inhalation of conidia. It commonly presents as localized, lymphocutaneous, disseminated, or systemic forms. The testicles are among the organs that can be affected, often manifesting as a testicular mass. This work is aimed at analyzing a clinical case along with a bibliographic review on the testicular involvement of sporotrichosis.

**Case Report:**

A 35-year-old male with positive HIV serology and a history of psychoactive substance use presented with disseminated ulcerated lesions that progressed over 1 month, with a positive blood culture for sporotrichosis. Upon hospitalization, a painless lump in the right testicle was diagnosed upon palpation and peripheral vascularization. An orchiectomy was performed, and anatomopathological analysis revealed the presence of *Sporothrix*.

**Discussion:**

Few reports on testicular sporotrichosis were found in the literature. Systemic forms are rare and are often associated with immunosuppression, particularly in cases of HIV and chronic alcoholism. This immunosuppression can favor the prevalence and dissemination of the fungus. The fungus also produces melanin, which aids in evading the immune system. The gold standard for diagnosis is culture. Furthermore, the treatment of choice is prolonged therapy with Amphotericin B, followed by itraconazole.

**Conclusion:**

Given the suspicion of disseminated sporotrichosis and the presence of a testicular nodule, the possibility of testicular sporotrichosis should be evaluated while maintaining attention to the differential diagnosis for neoplasia.

## 1. Introduction

Sporotrichosis is an endemic fungal infection in Latin America, especially in Brazil. Thermodimorphic fungi of the genus *Sporothrix* cause this disease [1]. Transmission occurs through traumatic inoculation during the handling of plants, soil, and/or organic matter containing fungal propagules and through inhalation of conidia during agricultural activities [2]. In Brazil, it is considered a zoonotic disease, with compulsory notification in some states of the country and is predominantly caused by *Sporothrix brasiliensis*. The state of Rio de Janeiro accounts for the largest number of cases in the last 20 years [3].

This disease has a subacute to chronic manifestation. It is classified according to the affected area into cutaneous and extracutaneous sporotrichosis. The extracutaneous form, also called systemic, involves organs and systems. Extracutaneous infections are classified according to the anatomic location of the lesion and the route of infection (primary or multifocal), with their clinical forms including pulmonary, osteoarticular, ocular, central nervous system, and mucosal involvement [4–6].

Systemic involvement, including cutaneous dissemination and testicular injury, is rarely described in the literature. The authors Espinoza-Hernández et al. [7] and Duani et al. [8] present two case reports of sporotrichosis also manifesting as a testicular mass, both in chronic alcoholic patients, diagnosed in vivo and postmortem, respectively. Radical orchiectomy was indicated in the first case. The objective was to analyze this clinical case alongside a bibliographic review on testicular involvement due to sporotrichosis. This case was structured in accordance with the CARE guidelines for case reports [9].

## 2. Case Description

Male, 35 years old, with social fragility, diagnosed with HIV 4 years before (current viral load 84,369 and CD4 count 257) and addiction to psychoactive substances, was admitted to the emergency unit of a university hospital due to asthenia associated with ulcerated skin lesions on the hands, back, and face, in addition to a lesion on the hard palate, at that time, with suspected fungal or neoplastic involvement. He complained of right orchialgia for 1 month, with the presence of a palpable, painless, manageable nodule in the upper testicular region, transitioning with the right epididymis, approximately 1 cm in size, and with no inguinal lymph node enlargement.

**Figure 1:**
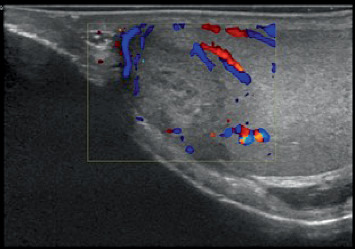
Heterogeneous lesion on ultrasound with a nodule of 2.3 × 1.4 × 1.3 cm in the right testicle.

Laboratory tests were requested to evaluate the testicular nodule, including B-HCG, alpha-fetoprotein, and LDH, all of which were within normal limits. Contrast-enhanced tomography of the abdomen and pelvis was performed, with no findings suggestive of lymph node enlargement. In the ultrasound with Doppler (Figure 1), a right testicular nodule (2.3 × 1.4 × 1.3 cm) with peripheral vascularization was found, raising the possibility of neoplasia during the examination.

**Figure 2:**
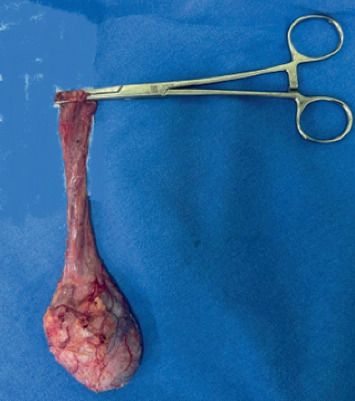
Orchiectomy of the right testicle, measuring 4.7 × 2.0 × 3.4 cm.

Due to the testicular nodule, where malignancy could not be ruled out, and considering the patient's unfeasible social conditions for follow-up, a surgical approach was chosen in a team meeting, involving right radical orchiectomy (Figure 2), a procedure performed without complications, with a macroscopically intact testicle, showing no masses in the albuginea. Concomitantly, biopsies of the skin and hard palate lesions were performed. The reports were simultaneous, showing morphologic structures compatible for sporotrichosis, in addition to a positive blood culture for *Sporothrix*. The cultures of the lesions were positive to *Sporothrix*.

The patient developed a surgical wound infection on the 3rd postoperative day, requiring wound drainage and antibiotic therapy guided by secretion culture (*Enterobacter* ESBL). He was discharged 30 days after surgery and returned for outpatient follow-up after 30 days, with satisfactory healing of the surgical wound, without complaints, and with a plan to continue the use of oral itraconazole for 6 months. Unfortunately, he was readmitted with central nervous system involvement due to the fungal infection, despite the antibiotics used at the time of medical discharge. The patient's clinical presentation was mental confusion, leading to admission to the emergency department. Neurological evaluation was performed through imaging, which suggested central nervous system involvement.

## 3. Discussion

Our literature research yielded 74 publications, of which 66 titles were fully reviewed (five papers were inaccessible) focusing on testicular involvement.  Table 1 describes the demographic and clinical details, as well as comments on the five studies included in the final selection. Four papers discussed disseminated clinical presentations, while one was a review of 39 other studies. In this review by Moreira et al., two studies involving testicular involvement were mentioned [5, 12].

Sporotrichosis was first described by Schenk in Baltimore, United States, and became an epidemic in Brazil in the late 1990s due to zoonotic transmission. Testicular sporotrichosis is a rare finding in the medical literature, typically occurring in association with severe systemic sporotrichosis and not as an isolated or incidental finding. Within the literature review conducted, there were two findings analyzed in cadavers [8, 10] and two findings in patients during clinical investigations for disseminated sporotrichosis [7, 11]. Among these, only Espinoza has a description of orchiectomy available.

The differential diagnosis with testicular neoplasms is inevitable in these cases, presenting a challenge for any clinician or urologist. In this reported case, the age and the description of the testicular mass on ultrasonography were decisive for the choice of surgical treatment. Rather than performing more conservative approaches such as biopsy or clinical treatment while awaiting a therapeutic response, the patient's clinical and social conditions also supported the decision for surgery. However, the patient was being investigated for possible fungal involvement at the time, due to skin and oropharyngeal lesions [11].

In our patient, postoperative complications included a surgical wound infection associated with a multidrug-resistant *Enterobacter* (ESBL), requiring drainage and targeted antibiotic therapy. Despite clinical improvement and adequate healing of the surgical wound, he later developed central nervous system involvement, a severe manifestation of disseminated sporotrichosis with high morbidity and mortality. These complications highlight the importance of close monitoring and long-term antifungal therapy in immunocompromised patients [6].

In individuals with HIV infection, sporotrichosis often assumes more aggressive and disseminated forms, with frequent pulmonary and central nervous system involvement, in addition to cutaneous and osteoarticular manifestations [7, 11]. The impaired cellular immune response, especially when CD4 counts are below 200 cells/*μ*L, predisposes these patients to atypical localizations and worse outcomes. Testicular involvement, although rare, appears more often in immunocompromised hosts, reinforcing the need for early suspicion and aggressive antifungal treatment in this subgroup.

Testicular sporotrichosis has no reported incidence or frequency, and there are no reports of bilateral testicular sporotrichosis. Among the four cases of testicular infection by sporotrichosis, ages ranged from 26 to 54 years. Two cases involved HIV infections without adequate treatment, and the other two were associated with significant alcohol consumption. There is also a case in the literature of a patient with right testicular edema and a diagnosis of sporotrichosis by biopsy, who showed improvement in the edema after starting clinical treatment, suggesting the possibility of conservative treatment in similar cases, despite their rarity [6].

The dissemination of *Sporothrix* occurs mainly by lymphatic and hematogenous routes, particularly in immunosuppressed patients, which explains the potential for involvement of deep organs such as the lungs, central nervous system, and testes [7, 8, 10, 11]. While the majority of cases remain cutaneous or lymphocutaneous, disseminated disease requires high clinical suspicion, especially in patients with HIV, alcoholism, or severe comorbidities. In this context, testicular sporotrichosis, although uncommon, should be considered in the differential diagnosis of testicular nodules, particularly when associated with disseminated fungal infection.

## 4. Conclusion

Although testicular presentation is rare, in cases suggestive of disseminated sporotrichosis or any other fungal etiology associated with suddenly appearing testicular nodules, we should maintain a high suspicion for the possibility of genital involvement. There is currently no established protocol or defined management for these cases in the literature. If there is persistent diagnostic uncertainty regarding the possibility of neoplasia, radical orchiectomy is a viable option; however, there are reports in the literature suggesting the possibility of disease regression with clinical treatment.

## Figures and Tables

**Table 1:** Summary of data from sporotrichosis with testicle involvement.

**First author, year, reference**	**Title**	**Country of origin**	**Clinical presentation**	**Age**	**Comorbidity—CD4 cell count (cell/mm** ^**3**^ **)**	**Therapy**	**Outcome**	**Comments**
Duani, 2019, [8]	Meningeal and multiorgan disseminated sporotrichosis: A case report and autopsy study	Brazil	Disseminated (skin, frontal and malar regions, arms legs, nasal septum, renal, hepatic, splenic, prostate, testicles and meningeal/CNS involvement)	36	Homeless, heavy drinker, drug abuser—negative serologies for HIV	AmB	Autopsy study—dead due to acute ischemic brain event with hemorrhagic transformation in the right frontal lobe	Case report Diagnosed by culture

Espinoza-Hernández, 2014, [7]	Disseminated sporotrichosis with cutaneous and testicular involvement	United States	Disseminated (skin, tenders, muscles, testes)	54	No previous comorbidity, only chronic drinker—negative serologies for HIV	AmB	Death	Case report Diagnosed by culture: *S. schenckii*

Silva-Vergara, 2005, [10]	Multifocal sporotrichosis with meningeal involvement in a patient with AIDS	Brazil	Disseminated (*Sporothrix schenckii* in meninges, lymph nodes, marrow bone, skin, testicles, epididymides, and pancreas)	29	AIDS—73	ITC primarily, then switched to AmBd	Death	Case report Diagnosed by culture: *S. schenckii*

Heller, 1991, [11]	Disseminated sporotrichosis in patients with AIDS case report and review of the literature	United States	Disseminated (cutaneous, bloodstream, eye, osteoarticular, bone, lung, and testes)	49	AIDS—11	Initially treated with AmBd (2350 mg, total dose) + 5-FC, then switched to ITC and SSKI	Death	Case report Diagnosed by culture: *S. schenckii*

Abbreviations: 5-FC, 5-flucytosine; AmB, Amphotericin B; ITC, itraconazole; SSKI, saturated solution of potassium iodide.

## Data Availability

The data that support the findings of this study are available from the corresponding author upon reasonable request.
