# Correction to “Systemic Sporotrichosis With Testicular Involvement: Literature Review and Case Report”

**DOI:** 10.1155/criu/9840325

**Published:** 2026-07-23

J. E. R. Rodriguez, A. N. L. Tuma, A. Iwamoto, et al., “Systemic Sporotrichosis With Testicular Involvement: Literature Review and Case Report,” *Case Reports in Urology* 2025, no. 1 (2025): 4235866, https://doi.org/10.1155/criu/4235866.

In the above article, the described patient was previously reported in another article [1].

As such, the first paragraph of “Section 2. Case Description:”

“Male, 35 years old, with social fragility, diagnosed with HIV 4 years before (current viral load 84,369 and CD4 count 257) and addiction to psychoactive substances, was admitted to the emergency unit of a university hospital due to asthenia associated with ulcerated skin lesions on the hands, back, and face, in addition to a lesion on the hard palate, at that time, with suspected fungal or neoplastic involvement. He complained of right orchialgia for 1 month, with the presence of a palpable, painless, manageable nodule in the upper testicular region, transitioning with the right epididymis, approximately 1 cm in size, and with no inguinal lymph node enlargement.”

Should read:

“A 35‐year‐old male with social fragility, diagnosed with HIV 4 years before (current viral load 84,369 and CD4 count 257) and addiction to psychoactive substances, was admitted to the emergency unit of a university hospital due to asthenia associated with ulcerated skin lesions on the hands, back, and face, in addition to a lesion on the hard palate, at that time, with suspected fungal or neoplastic involvement. He complained of right orchialgia for 1 month, with the presence of a palpable, painless, manageable nodule in the upper testicular region, transitioning with the right epididymis, approximately 1 cm in size, and with no inguinal lymph node enlargement. This patient was originally reported by Cognialli et al. [1].”

Furthermore, Figure 1 was published in the prior article and has not been attributed. The correct Figure 1 and Figure 1 legend are as follows:

**Figure 1:**
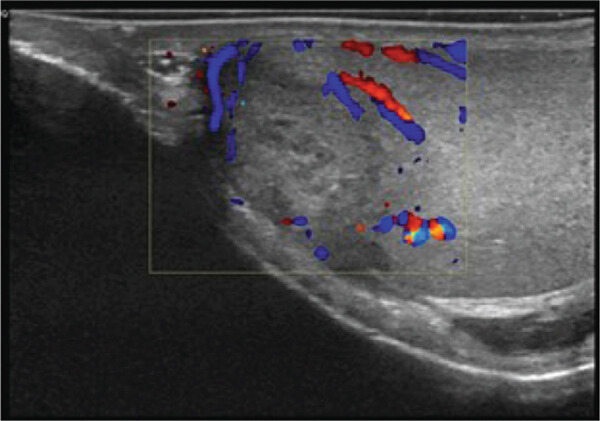
Heterogeneous lesion on ultrasound with a nodule of 2.3 × 1.4 × 1.3 cm in the right testicle [1].

We apologize for this error.
